# *Weaving Lines of Inquiry*: Promoting Transdisciplinarity as a Distinctive Way of Undertaking Sport Science Research

**DOI:** 10.1186/s40798-021-00347-1

**Published:** 2021-08-03

**Authors:** Carl T. Woods, James Rudd, Duarte Araújo, James Vaughan, Keith Davids

**Affiliations:** 1 Institute for Health and Sport, Victoria University, Melbourne, Australia; 2 Norwegian School of Sport Sciences, Oslo, Norway; 3 CIPER, Faculdade de Motricidade Humana, Universidade de Lisboa, Lisbon, Portugal; 4 AIK Football, Research & Development Department, Stockholm, Sweden; 5 School of Human Movement and Nutritional Sciences, The University of Queensland, Brisbane, Australia; 6 Sport & Human Performance Research Group, Sheffield Hallam University, Sheffield, UK

**Keywords:** Team science, Knowledge, Innovation, Translation, Disciplinisation, Transdisciplinary

## Abstract

The promotion of inter- and multidisciplinarity — broadly drawing on other disciplines to help collaboratively answer important questions to the field — has been an important goal for many professional development organisations, universities, and research institutes in sport science. While welcoming collaboration, this opinion piece discusses the value of *transdisciplinary* research for sports science. The reason for this is that inter- and multidisciplinary research are still bound by disciplinary convention — often leading sport science researchers to study *about* a phenomenon based on pre-determined disciplinary ways of conceptualising, measuring, and doing. In contrast, transdisciplinary research promotes contextualised study *with* a phenomenon, like sport, unbound by disciplinary confines. It includes a more narrative and abductive way of performing research, with this abduction likely opening new *lines of inquiry* for attentive researchers to follow. It is in the *weaving* of these lines where researchers can encounter new information, growing knowledge in-between, through, and beyond the disciplines to progressively *entangle* novel and innovative insights related to a phenomenon or topic of interest. To guide innovation and the development of such research programmes in sport science, we lean on the four cornerstones of transdisciplinarity proposed by Alfonso Montuori, exemplifying what they could mean for such research programmes in sport science.

## Key Points

- This current opinion promotes transdisciplinarity as a distinctive way of conceptualising and engaging with empirical inquiry in sport science.
- We argue for a view of transdisciplinarity which would take sport science researchers *in-between*, *through*, and *beyond* disciplinary boundaries when seeking to explain complex phenomena.
- To guide sport science researchers adopting such an approach to empirical inquiry, we elaborate on the four cornerstones of transdisciplinary research proposed by Alfonso Montuori, threading through examples as to what they could look like for such research programmes within sport science.

## Prologue: ‘Stepping out’

"yet as biologists gaze into the mirror of nature, what they see – reflected back in the morphology and behaviour of organisms – *is their own reason*" Tim Ingold ([1], p.19, emphasis added)

For those who primarily dwell in academic institutions, the words ‘multidisciplinary’ and ‘interdisciplinary’ would be somewhat familiar, fast becoming staple additions to grant or funding applications. Such words are intended to promote the development of collaborative research programmes, where problems are viewed simultaneously from either different disciplinary perspectives or disciplinary knowledge, manifest in methodologies, tools, or concepts, are transferred to address important questions in a certain field. This additive approach to research is believed to progress disciplinary knowledge — encouraging researchers to look at problems through different disciplinary lenses — helping them ‘see’ things that may otherwise have been hidden [2].

In sport and physical education, at least, this integrative theme to research is not overly contemporary. For many years, the vision of the European College of Sport Science — primarily focused on professional development of sport and exercise scientists — has referred to the promotion and application of inter- and multidisciplinary approaches to the discipline (https://sport-science.org/index.php/home/our-vision). Additionally, in a special issue of Quest, published over 30 years ago, Newell and Rovegno [3] called for research in physical education to broaden its horizons, stating that ‘[…] physical education requires dual efforts of motor learning researchers *and* physical educators if the knowledge base in this area is to advance’ (emphasis added, p. 187). To us, what these authors were suggesting was not necessarily multi- or inter-, but an approach to scientific inquiry better reflective of *trans*disciplinarity. That is, contextualised research that horizontally (i.e., ‘dual efforts’) weaves empirical (i.e., ‘motor learning researchers’) and experiential (i.e., ‘physical educators’) knowledge into an entanglement of threads to explain a phenomenon, in context. This approach would take applied scientists and practitioners beyond where they could go by preferencing one source of knowledge alone [4]. It differs from multi- and interdisciplinarity by not just viewing problems from a range of different disciplinary perspectives, nor by simply transferring methods from one to another discipline. Rather, as we discuss here, transdisciplinarity offers a distinctive way of thinking about, and engaging with, scientific inquiry [5]. In particular, it offers a way of illuminating the entanglements of real-world and wicked challenges that transcend disciplinary boundaries. To this end, there is a growing recognition that while a researcher may start their inquiry (or even academic career) from a certain disciplinary vantage point (coupled with their own paradigmatic assumptions, acculturated in a specific time and place), a transdisciplinary approach encourages them to remove their disciplinary blinkers, *step out*, and engage directly with the phenomenon, opening unique lines of inquiry that could transcend current ways of doing [2].

Despite the prompting of Newell and Rovegno [3], few in sport and physical education have accepted their challenge and attempted to broaden the horizons of their research (for notable exceptions, see [6–8]). Specifically, sport science has largely been criticised for its confined and insular thinking. The majority of scientists still seek to investigate sport problems in silos through mono-disciplinised lenses, labelled as ‘biomechanists’, ‘physiologists’, or ‘psychologists’ (for critiques, see [5, 7–10]). This disciplinisation in sport is perpetuated through the continued specialisation of scientific journals that clearly draw boundaries around a (sub-)discipline in which the ‘research topic’ is considered to ‘belong’. It is, however, important to recognise that disciplinary approaches can and have produced insightful findings [11]. But adopting an isolated, disciplinised approach to scientific inquiry when seeking to understand complex phenomena does risk limiting understanding, precisely through specialisation — contained within a pre-defined scope or boundary of inquiry. This means that, at best, mono-disciplinary approaches can offer a partial view of a complex, entangled phenomenon, but at worst, this partial view is conflated to represent functioning of the whole [12]. This is an issue of relevance for underpinning theory and practice in sport science [13], as ‘unlike academic disciplines, life does not break down into neat categories and disciplines, and we ignore them [i.e., relations] at our own risk’ ([11], p. 46).

In this opinion piece, we consider transdisciplinarity as a distinctive way of thinking about, and undertaking, research in sport science. We first discuss what transdisciplinarity is, drawing reference to it being a mode of engaging in scientific inquiry that extends *in-between*, *through*, and *beyond* the disciplines. Then, guided by the work of Alfonso Montuori (e.g. [11, 14, 15]), we explore four main dimensions — cornerstones — of transdisciplinarity, exemplifying their implications for the development of such a research programme in sport science.

## The Homeless Scholar

### Wayfinding Through Knowledge Landscapes

We start our journey with a brief introduction to the key ideas of an inspirationally unique scholar, Tim Ingold. From a strict disciplinary perspective, he is classified as an anthropologist — going even further — a social anthropologist. But this academic placement offers little insight as to the type of scholar he is. For example, among others, he has significantly contributed to discussions on the topics of education [16], archaeology and art [17], perception of the environment [1], and technology and skill [17, 18]. To adopt a rather Batesonian-inspired phrase, Ingold could thus be viewed as being intellectually *homeless* [19]. Meaning, his inquiry is not particularly wedded to a specific discipline and, by default, locked into disciplinary ways of doing or being. There is an important caveat here, however, which is that Ingold’s contributions to these topics are not within the topics themselves, but in their *relatedness* to each and anthropology (emphasised eloquently in his exceptional book, *Making: Anthropology, Archaeology, Art and Architecture* [20]). Simply, it is in the *weaving* together of these topics where he has looked to grow knowledge toward a richer and more holistic understanding of human behaviour — progressing anthropology beyond its traditional, dualistic disciplinary roots. It is in this weaving where he avoids the disciplinary fragmentation that could result from studying each topic separately — in isolation — and in doing so, opens new, unique, and significantly richer *lines of inquiry* to follow.

While not explicitly referencing himself as transdisciplinary, Ingold does emphasise being ‘anti-disciplinary interdisciplinary’ ([16], p. 74), implying a way of being that is not confined to disciplinary convention, but one that situates exploration, an ethos of amateurism, mystery, collaboration, reflection, and embracement of the unknown at its core. These ideas underpin an approach to scientific inquiry that Ingold [21] refers to as ‘obviation’; a notion brought forward to contrast the ‘complementarity’ he argues is common to many Westernised, socially scientific programmes that study human behaviour^1^; stating:

I propose that we consider humans as indistinguishable organisms and persons, participating not in two worlds but in one, consisting of the entire field of their environmental relations. (p. 48)

Such a holistic perspective regarding scientific inquiry encourages researchers to explore beyond disciplinary walls to blend seemingly disparate sources of knowledge ‘to create something new that is irreducible to the disciplinary components that were initially brought to bear’ ([22], p. 31). It is, however, a rather different approach to scientific inquiry — a sentiment noted by any budding postgraduate researcher who may be asked to ‘find and tick a box’ on an application form that specifies the discipline in which their project is to ‘fit’. We believe this dominant view of ‘academic disciplines’ highlights a systemic issue related to the structure of the modern university. These institutions typically promote the compartmentalisation of knowledge through continued disciplinary fragmentation and specialisation [16, 23]. This is an issue because disciplinary specialisation risks replacing interactive and relational thinking with reduced and disjunctive thinking, leading researchers to study *about* a phenomenon removed from its ecology of relations [14]. These insights advocate adoption of a systemic perspective for studying phenomena that are inherently nested and embedded, and may facilitate the weaving of an entanglement through a broadened landscape of inquiry. Such relational perspectives have been articulated in different forms over many decades. For example, in highlighting the role of dynamical systems theory in modelling complex phenomena, the mathematician Henri Poincaré ([24], p. xxiv) suggested that the focus of science should not be to collect data about ‘things’, arguing that: ‘the aim of science is not things themselves […] but the relations among things; outside those relations, there is no reality knowable’.

This perspective, importantly, leads us to *the promise of transdisciplinarity *— an embedded, contextualised systems orientation to scientific inquiry that foregrounds the reciprocity of the inquirer (researcher) and inquiry (research) [2].

## Citizens of Everywhere

### In-between, Through, and Beyond Disciplinary Boundaries

Indeed, the homeless scholar portrayed here is that of the transdisciplinary researcher — an academic wayfinder who follows lines of inquiry that often takes them *in-between*, *through*, and *beyond* disciplinary boundaries [25, 26], weaving together the lines of inquiry as they go [27]. From a transdisciplinary perspective, knowledge is understood relationally, as something entangled that forms a larger, holistic meshwork of ideas [11]. Given the constant re-organisation of knowledge when understood in this way, ‘trans’ can be viewed in a *trans*itory way, meaning researchers move *with* the inquiry as it opens up, zooming in (i.e., observing local interactions) and out (i.e., appreciating sociocultural factors) as they zigzag through what are traditionally considered disciplinary landscape boundaries to enrich their understanding of a particular topic. To ‘know’, in this sense, is then *to move*.

This description of transdisciplinarity has been shaped by many scholars, like Jack Lee Mahan [28]. Specifically, in his doctoral dissertation, *Toward Transdisciplinary Inquiry in the Humane Sciences*, he emphasised that the interaction between increasingly specialised research would need more than multi- or interdisciplinary approaches^2^, potentially requiring the *blurring* of disciplinary lines:

An earnest attempt to ameliorate personal and social human problems, and simultaneously affect a concerned humane science, requires methods, concerns and foci of inquiry which *transcend* and *supplement* traditional boundaries of academic domain ([28], p. 7, emphasis added)

In other words, transdisciplinary research is characterised by a common trend to transcend disciplinary landscape boundaries, bringing continuity (and perhaps unity) to inquiry and knowledge [26, 28]. There is an important point to briefly highlight here regarding the word ‘landscape’, which is that conventional connotations of this word view the suffice ‘scape’ as ‘scopic’, meaning ‘land-to-be-looked-at’ [29]. Olwig [30], however, reminds us of its etymology (Germanic), *landshaft*, meaning ‘land-being-shaped’. Understood in this more ecological way, as the transdisciplinary researcher wayfinds in-between, through, and beyond disciplinary boundaries, they concurrently shape the land*scape* through their weaving, *and it shapes them*, which means knowledge is ongoing, temporal, and transformative [20].

This disciplinary transcendence is important for a few reasons. First, it encourages researchers to eschew dominating, intra-paradigmatic assumptions often engrained, or hidden, within disciplinary ways of doing [11]. Sport science research, for example, is founded on typically quantitative paradigms, detected in phrases such as ‘data are power’ in academic publications that seem to conflate the hypothetico-deductive theory of scientific method as ‘the’ way of undertaking research and gaining knowledge [31]. Such disciplinary assumptions can considerably limit the growth of knowledge that can occur by adopting different, meta-paradigmatic assumptions, like interpretivism when exploring the role of constraints on performance and the development of sporting expertise [32]. This is why multi- and interdisciplinarity can still be limiting, in that these approaches may be used in an additive way that rarely questions underlying disciplinary paradigms (or roots) — instead viewing problems at a more methodological level of analysis [14].

This common fixation within sport science on *analysis* (i.e., move near and deconstruct to understand) at the expense of *synthesis* (i.e., step back and weave together to understand) [33] is reflective of another paradigmatic assumption, one founded on a reductionistic ontology that sets its unit of analysis almost exclusively on the organism, or even its subsystems (e.g. cellular, molecular, neuronal). This, however, is not exclusive to sport science, with Costanza [34] arguing that most university researcher training and education programmes conflate analysis over synthesis, creating an unbalanced bias between the two, which inadvertently drives a dualism. This bias can cause limitations in scientific research because scientists risk focusing nearly all of their time on the collection and analysis of data. This restriction could come at the expense of critically examining what the data ‘mean’ or how they can be integrated to effectively support practice and positively shape community interaction within an ecosystem [34]. Further, a dualistic or reductionist unit of analysis can exacerbate *organismic asymmetries*, in that the internal processes of an organism (i.e., athlete, performer, coach) are positioned as hierarchical to, and studied detached from, the environments (inclusive of historical contexts) which they inhabit (see Davids and Araújo [35] for a detailed overview).

Disciplinary transcendence also brings the phenomenon of interest to the core of the research programme — rooting it in *context* [11]. What this means is that research becomes topical, as opposed to disciplinary [2], encouraging the researcher to engage directly with phenomena in situ. In sport science, this view would see the researcher become entangled *with* athletes, coaches, support practitioners, and other stakeholders, collaboratively working together to pose and respond to questions that open new lines of inquiry to be followed [32]. This collective embeddedness leads to a more holistic understanding of a phenomenon (as it is more than a sum of its parts). Given this, transdisciplinary research is more *abductive* [36], contrasting with *deductive* or *inductive*, leading groups of researchers into knowledge regions they may not have initially planned to go to, individually [37]. Elsewhere, we have argued that such an approach to scientific inquiry demands responsivity, with researchers attending to new information as the process of inquiry unfolds to study the emergence of behaviours in real-time [27]. Extending our ideas, this responsivity can be understood as a type of submission. Here, attentive and responsive researchers, open to various possibilities, wait propitiously on circumstances to emerge through their inquiry, following them up to deepen their understanding of a topic or phenomenon. Thus, transdisciplinary researchers do not impose disciplinary conventions *onto* a topic or phenomenon, but *actively follow* pertinent sources of information that emerge to understand ‘it’ as it really is. Indeed, this approach makes it difficult to map out in advance a specific route intended to solve a problem — manifest in doctoral research programmes that demand pre-planned proposals within, for example, six months of enrolment to ‘confirm candidature’. Indeed, as eloquently stated by Ingold ([25], p. 138), ‘to sail the seas — is to cast off into the stream of a world in becoming, with no knowing what will transpire. It is risky business’. But for the transdisciplinary researcher, this risk, uncertainty, and submission is embraced, enabling them to grow and enrich knowledge beyond pre-determined disciplinary conventions or mapped out paths, potentially even leading to the emergence of entirely different ways of knowing and doing that are yet to be conceived [27].

To elaborate, we next discuss four cornerstones of transdisciplinary research as proposed by Alfonso Montuori [11, 14, 15]: (1) *inquiry-based, not disciplinary-based*, (2) adopting a *complex systems perspective*, (3) *studying with, not about* — situating the inquirer within the inquiry, and (4) *meta-paradigmatic, not intra-paradigmatic*. These cornerstones are intended to offer researchers in sport science with a basis in which to approach transdisciplinary inquiry. To support this aim, we thread through examples of what they could mean for such research programmes.

## The Cornerstones of Transdisciplinarity

### Inquiry-Based, Not Disciplinary-Based

The first cornerstone of transdisciplinarity, as suggested by Montuori [11, 14, 15], is to situate the inquiry at the core of the research programme, not the discipline. What this means is that *topical* questions emerge from the interests and concerns of researchers, often guided by their own experiences developed from being embedded in the subject context. This is different from traditional approaches to research, which tend to be pre-determined and framed by disciplinary ‘norms’, ‘agenda’, or ‘conventions’, or in the case of postgraduate students, perhaps even by the beliefs and interests of an advisor, established before a phenomenon is studied. The problem with disciplinary-based research, though, is not that it is *not* informative, but that it risks reductionism, offering multiple, partial, or worse, non-representative views of a phenomenon, which could then be conflated as being a perspective of ‘the whole’ [11]. For example, sport scientists labelled as ‘physiologists’ may study the efficacy of practice task design, driven by specificity of *physiological* responses measured relative to those observed in competition. Those labelled ‘biomechanists’ may study the same topic, recording the specificity of *biomechanical* variables relative to values observed from athletes in competition. Undoubtedly, both disciplines may uncover interesting facts about the efficacy of the practice task design, but viewed in isolation and removed from context. In other words, they clearly only provide a partial view *about* the phenomena. The challenge for transdisciplinary researchers, then, is to identify and engage with the pertinent sources of knowledge that guide the inquiry, weaving them together as they explore the vast regions in-between, through, and beyond the disciplinary landscape.

To assist with this challenging task, Montuori [15] proposes that researchers start their inquiry, not through a disciplinary lens (i.e., as a ‘physiologist’ or ‘biomechanist’) but by developing a rich narrative of the phenomenon of interest by dwelling within ‘it’, responding to ‘it’, and attending to ‘it’. Based on the nature of the knowledge and information that emerges, through this seemingly ethnographic process, the researcher starts to identify key lines of inquiry that require following up, actively looking for their *relatedness* to form a more holistic — entangled — understanding of the phenomenon. Discussion and joint reflection among researchers can be an important component of this first cornerstone, encouraging researchers to routinely pose questions, like:

- ‘What is the phenomenon we are trying to better understand and what appears to be important in understanding it?’
- ‘How does this newly encountered information relate to the phenomenon?’
- ‘How does this newly encountered information relate to or correspond with other lines of inquiry we have uncovered?’
- ‘Using this newly encountered information, where should we explore next?’

Note that there are no restricted areas roped off by disciplinary ways of doing in this inquiry-based approach. This is why researchers are likely to be zooming in and out, zigzagging across different disciplines and sources of knowledge, developing an overview on how newly encountered information may relate to the phenomenon of interest. This approach does not mean that researchers need to become content ‘experts’ in each knowledge region encountered in the landscape, but rather team-based ‘comprehensivists’ [15], growing their knowledge of a plurality of disciplines and how such disciplines can be used to explain features, or parts, of the phenomenon of interest. Metaphorically, they could be seen to be *pitching tents* in various regions as they attend to the inquiry in its unfolding. This reflection, therefore, can help the researchers continually (re)orient themselves when following up lines of inquiry that emerge — viewed somewhat like a northern or southern constellation that guides their exploratory journey. However, a critical point should be made here, which is that such a narrative approach is not the end of the transdisciplinary scientific inquiry. Rather, this is an *initial approach* that intends to help researchers ‘set the scene’, supporting them in the development of key eco-physical variables that can be mathematically modelled to describe and explain system behaviour in a contextualised way. For an extensive overview on the development and modelling of such variables (viewed like order parameters in dynamical systems theorising) within sport science, we nudge readers toward the work of Araújo and Davids [38].

### Complex Systems Conceptualisation

According to Montuori [14], individuals are traditionally taught to organise, manage, and structure their knowledge into digestible ‘pieces’, established through *disjunctive* and *reductive* thinking. These chunks of information are then progressively put together, in an additive way, which are intended to help an individual ‘know about the whole’ [39]. Indeed, structuring knowledge in this way can be effective, as it allows individuals to deduce reasoning based on a select number of variables [11]. Progressively, though, this disjunctive approach to organising and structuring knowledge has led to the emergence and solidification of disciplinary specialisation, inadvertently framing how individuals conceptualise phenomena: confined to the discipline in which it is viewed from, often without a unified picture able to inform real world applications. This unidimensional perspective can be limiting, especially when viewing complex phenomena (composed of many interacting parts and nested sub-systems). Since knowledge is split off or fragmented into parts, individuals may ((un)wittingly) ‘ignore’ information that does not fit within their disciplinary specialisation, regardless of what it can offer toward the explanation of the broader phenomenon of interest [11].

In contrast to this traditional way of structuring and organising knowledge, transdisciplinarity encourages researchers to think differently about a phenomenon, guided by theories of complexity and non-linearity, as opposed to reductivity and linearity [15]. This perspective change is surmised by Morin and Kern ([40], p. 130), who state that:

We need a kind of thinking that relinks that which is disjointed and compartmentalised, that respects diversity as it recognises unity, and that tries to discern interdependencies.

This type of thinking, exemplified by Poincaré’s [24] dynamical systems perspective, demands that phenomena of interest be understood through their broader ecology of relations, with researchers progressively learning how such relations interact to form the whole. By adopting complexity in their inquiry, researchers progressively understand how other disciplinary and/or societal ways of being and doing may be useful in contributing to explanations of a phenomenon in a more abductive way, encouraging them to structure their knowledge, based on what is pertinent.

To help guide this complexity-embracing and holistic perspective for sport science researchers, they could root their inquiry — their observations — in a theoretical framework which is powerful and rich enough to frame understanding and methods from numerous sub-disciplines. This could provide support ranging from practical applications to theoretical modelling and empirically investigating relevant phenomena. Following the ideas of Rothwell et al. [5], we suggest that the framework of ecological dynamics (a transdisciplinary framework for studying sport performance and learning, in and of itself) could be a particularly fertile playing field for transdisciplinary research in sport science [41, 42], based around four of its key components:

1. Its roots in complexity sciences [43, 44] encourage sport scientists to consider performance holistically — appreciating how the interaction of athletes, coaches, support staff, organisational stakeholders, supporter-bases, environmental factors like geographical features and locations, and socio cultural norms (for example) shapes emergent behaviours. Indeed, different layers of these interactions may be more pertinent than others, based on the inquiry, like investigating how practice tasks are designed compared to how a country views its national talent development programme. Both issues, though, would still require consideration of diverse interacting parts (which may clearly still entangle at different levels).
2. Given the influence of complexity sciences, behaviour is viewed in a non-linear phenomenon [45], meaning that, due to interconnectivity, small, subtle changes in system properties can have large, disproportionate effects over varying timescales of performance, learning, and development, and sometimes little effect. For example, subtle changes in a child’s tennis racquet (i.e., its grip, racquet head size, and/or weight) may lead to the emergence of qualitatively different shots played when compared to playing with a larger, adult-scaled racquet [46].
3. The most insightful unit of analysis is at the level of individual-environment interactions [47], meaning that (athlete, coach, team, organisational) behaviour cannot be understood separate from the context or environment in which it emerges (ideas founded on the theory of affordances by James Gibson [48]). This is particularly important for sport science research, as it is traditionally rooted in a reductionistic and deterministic ontology, leading to *organismic asymmetries* in research and practice, as behaviour is viewed separate from its ecology of relations [35].
4. Given this unit of analysis, emergent system behaviour is predicated on interacting (performer, task, and environmental) constraints [49]. The type of shot played by a cricket batter, for example, would be conceptualised not just as a discrete ‘action’, but as an emergent property to satisfy key, interacting (emerging and decaying) constraints — such as the action capabilities of the batter, the bounce of the ball off the wicket, the position of fielders, the ever-changing scoreline, the format of the game (test, 20-20 or 1-day), the local weather conditions, and the norms of the country in which the batter is based.

Indeed, complexity thinking and organising one’s knowledge in a way that enables them to identify the pertinent interactions between the various lines of inquiry that emerge can be most challenging. This difficulty can be compounded if researchers are removed from the context in which the research is taking place, leading us to the third cornerstone of transdisciplinary research.

### Studying with, Not About: Situating the Inquirer Within the Inquiry

To develop a detailed understanding of the relations between the lines of inquiry that open when viewing phenomena in a complex system, transdisciplinarity calls for researchers to be situated *in* the research [11]. This integration is deeper than simply mastering the relevant literature viewed from the outside, but requires researchers to be embedded into the inquiry so they can *engage* with the pertinent sources of information that emerge as the inquiry unfolds [11]. In other words, ‘[i]n the pursuit of truth, [transdisciplinary] research is as much about the discovery of questions *in* practice as about the answering of them *by way* of practice, and the former continually overflows the latter’ ([16], p. 74). This means that researchers ask questions of the subject matter (both verbally and non-verbally — perhaps through carefully manipulating environmental features); watching, listening, and feeling how it responds, waiting on it to progressively show the important information, while understanding how this information relates to other aspects of the inquiry. Further, and thinking creatively, given that transdisciplinarity calls for researchers to engage with sources of information, they may even move beyond simply reading relevant literature about the topic of interest (i.e., in published articles, books, or book chapters), and reach out to the authors of such work (if possible). In addition to the immediate subject matter, researchers could ask questions of the authors, helping them to understand the authors’ intentions for studying a similar topic. This engagement may lead to important resolutions with regard to the current phenomenon of interest that are unable to be known by simply reading about it in published works — thereby opening new lines of inquiry to be woven together in a form of *correspondence* [20].

Transdisciplinarity calls for this deeper engagement between the inquirer and inquiry because it is rooted in daily activities, meaning that knowledge is not abstracted, but is continually contextualised within a broader entanglement of relations, inclusive of the researchers’ own perspectives when engaging with the inquiry [11]. This is captured by Montuori ([14], p. 7), stating that:

Such an approach recognizes the lived experience and subjectivity of the inquirer, the person reading the book who then hopes to put some of this material into practice. The lived experience occurs in a context, in a network of relationships, in an ecology.

This more ecological (embedded) perspective differs from traditional approaches to scientific inquiry, which seek to remove researchers from the research (heightening ‘objectivity’), positioning them ‘above’ a phenomenon to simply observe (through disciplinised lenses) — thereby, studying and documenting *about* it. Comparatively, a transdisciplinary approach encourages transparency, where the researchers’ perspective is constantly brought to the front through self-inquiry and self-assessment, routinely challenging them to question their own paradigmatic assumptions [11]. By doing so, researchers are able to ‘step out’ (into context) and embrace the phenomenon as it exists, studying *with* it, attending to its rhythms with clear eyes, tuned ears, and a receptive touch.

This more contextualised approach could signify that a research programme in sport science requires researchers to be deeply embedded in a performance setting or organisation when seeking to study a particular topic. How practice tasks are designed to support skill development in ice climbing, for example, would require researchers to engage *with* the community of athletes, coaches, support staff, and other stakeholders. Gradually, they would develop a rich narrative of ice climbing to progress their inquiry. Such an ethnographic approach could help them uncover many interacting and entangled parts that shape why practice tasks are designed the way they are — perhaps even noted by actually engaging in the activity of ice climbing. This constant interaction would allow them the opportunity to consider pertinent eco-physical variables to be modelled in the explanation of complex, interacting features of the ice climber-environment relation. For example, a practice task may be designed based on the entanglement of the relationships formed between athletes and coaches, as well as by the traditional socio cultural constraints that shape ‘how’ the sport is played or activity is ‘done’ within a particular country, perhaps preferencing creativity and flair, or control and physicality (for a detailed example see [50]). This approach helps the researcher contextualise the inquiry [33], blending experiential and empirical knowledge to develop a richer understanding of how practice tasks are designed to support skill development. More than this, though, transdisciplinary researchers see themselves as another thread in the broader entanglement of interactions, acknowledging that their underlying assumptions shape how *knowledge of* practice task design is being developed. Researchers do this by continually asking reflective questions that help them recognise how their own paradigmatic assumptions are implicating engagement with the inquiry — *Why did we ask the player/coach/support staff/organisational stakeholder the question we did? What led us to go down that line of questioning? Was the way we asked that question rooted in some assumption we are being influenced by? Do we think this new information is pertinent based on our own assumptions of how practice tasks could be designed?* Simply, transdisciplinary researchers are not detached from the inquiry, but deeply *entangled with it*. This is not something to be extricated, dampened, or shied away from, as indeed, researchers are part of the context in which they are seeking to ‘know’ (for an example of this type of participant observation, see Moeran [51]). It does, however, require constant collective reflection and transparency to recognise how underlying assumptions may be influencing what is known^3^—leading us to the fourth cornerstone of transdisciplinarity.

### Meta-paradigmatic, Not Intra-paradigmatic

Most disciplinary approaches to research are intra-paradigmatic, with researchers embedded in disciplinary paradigms — (un)willingly taking them as ‘the’ way of undertaking research [11]. The sporting biomechanist, for example, may rarely consider how socio cultural constraints shape the coordination of segment properties observed during a footballer’s kicking action. The strength and conditioning scientist may rarely consider what opportunities the rugby player attunes to during competition in order to ‘use’ their conditioning to continually (re)organise actions. Both simply go about their research while remaining within their quantitative, reductionistic paradigms — perhaps considering ‘outside’ factors, like those noted, as interesting things that reside beyond their disciplinary scope. This fourth cornerstone of transdisciplinarity, therefore, implicates the growth of knowledge — more specifically, how researchers come to know the things they research [11].

As mentioned throughout, disciplinary approaches rooted in intra-paradigmatic assumptions do have a role to play in applied science. But it is important to highlight their insularity when contrasted with transdisciplinarity, which draws on a plurality of disciplines and understanding of multiple ways of being to explain complex phenomena [11]. In other words, “[a]long with scholars who specialize, we also need scholars who ‘weave together’ what exists within disciplines, as well as related works in other disciplines, so that it can be applied to real world issues” ([53], p. 412). This plurality, though, is deeper than the transference of methodologies or disciplinary ways of doing, but extends to the appreciation of the underlying, rooted, paradigmatic assumptions of a discipline or community through which the researcher is wayfinding. Transdisciplinarity, thus, is *meta-paradigmatic* as opposed to intra-paradigmatic.

Consequently, transdisciplinary researchers weave together seemingly disparate disciplines, perspectives, or societal ways of being to guide their inquiry. For example, our transdisciplinary research on sports innovation has led us through seemingly disparate paradigms within social anthropology, ecological psychology, and sport science. This meta-paradigmatic approach allowed us to weave together multiple lines of inquiry relating to hunter-gatherer ways of being and sports organisational functioning to propose a model of knowledge growth that would take individuals beyond the confines of sport science, encouraging them to dwell in vastly different regions of the knowledge landscape in the continued search for innovative ways of doing (for a detailed insight, see [27]). Accordingly, transdisciplinarity requires the researcher to be creative when engaging in inquiry [53], weaving together paradigms, ideas, and ways of being and doing to create something that transcends disciplinary origins — shaping a path through a land*scape* that may not yet exist, but which can progressively offer a richer, more holistic view of a complex phenomenon.

## Concluding Remarks

Transdisciplinarity is an approach to scientific inquiry that encourages researchers to step outside of their ‘roped off’ disciplinary boundaries, and set sail in-between, through, and beyond the disciplines to weave together seemingly disparate sources of knowledge, skills, and experiences. Through the entanglements of such, deeper and significantly richer insights into a complex phenomenon can be sought. After all, in sport science, *context is everything*. As contended throughout this paper, contextualisation is a responsive way of undertaking research. It requires the researcher to submit to the phenomenon, yet maintain attentiveness to diverse and pertinent sources of information that emerge as they seek to understand a topic in context.

To support such research programmes in sport science, we leant on the seminal work of Alfonso Montuori [11, 14, 15], unpacking four of the main dimensions, or cornerstones, of transdisciplinarity. While indeed, it is our opinion that sport science needs more ‘weavers’ in research, it would be remiss if we did not highlight some of the difficulties in approaching scientific inquiry in this transdisciplinary way. First, the structural organisation and market orientation of the modern university promotes the compartmentalisation and commercialised specialisation of knowledge [23], making it challenging for researchers within sport science to blur disciplinary lines. Because of this, undergraduate and postgraduate students may be inadvertently educated in compartmentalised ways, making it difficult for them to adopt a complex systems perspective when studying a phenomenon. Second, the pressure on researchers to stick to disciplinary paths well-travelled to gain publications or funding (i.e., a track record) is noted. This calls for science within the sport to replace the empiricist compulsion to simply judge performance with traditional metrics and citations, with one that is founded on the celebration of a genuine sense of mystery and an embracement of the unknown — encouraging *people* to collaboratively work together based on what they may bring, venturing off the beaten track to find new sources of pertinent information that progresses understanding of a topic. So, while promoting transdisciplinarity as a different way of undertaking research in sport science, we appreciate that this challenging approach needs further, continuous development, and organisational and structural change, for it to be taken up more widely by researchers.

### Epilogue: ‘The Perpetual Traveller and the Unceasing Search for Knowledge’

To conclude, we return to the proposition of the homeless scholar — pursuing blurred lines of inquiry — that we introduced earlier in this paper. While we find the sentiment appropriate for the portrayal of the transdisciplinary researcher, in true transdisciplinary style, we would like to add our own, creative perspectives to it. To us, the transdisciplinary researcher is akin to a *perpetual traveller*, an individual who does not necessarily follow paths laid out in advance, but one who studies *for the love of the topic*. These are individuals who do not divide ‘professional’ from ‘personal’ life, but embody what it is that holds their attention. They embrace an uncertainty about the world as they constantly wayfind through various knowledge landscapes, weaving interesting and relevant lines of inquiry together as they dwell within the places encountered along their journey. These places, though, are not destinations — preconceived ends — but are rather waystations, punctuating an unceasing journey, each growing the traveller’s knowledge of the complex landscape as they find their way through it.

## Data Availability

Not applicable

## Footnotes

1 While we do not dwell on these approaches here, we do offer a definition — *complementarity* refers to ‘every aspect as a distinct, substantive component of being. It admits that the study of each component is bound to yield only a partial account’ ([21], p. 23). Contrastingly, *obviation* ‘is intent of doing away with boundaries by which these components have been distinguished. It claims that the human being is not a composite entity made up of separable but mutually complementary parts, such as body, mind and culture, but rather a singular locus of creative growth within a continually unfolding field of relationships’ ([21], p. 23).

2 It is important to briefly differentiate multi- and interdisciplinarity. Multidisciplinarity promotes the study of a topic, question or phenomenon by multiple disciplines at the same time. This typically entails disciplinary juxtaposition, where a team of researchers add together their disciplinary findings toward the solving of a problem. Interdisciplinarity, comparatively, brings together key components of multiple disciplines into a single programme of research. It typically involves the blending of disciplinary methods, used to frame complex problems. For further reading on these different approaches, we encourage readers to explore the work of Songca [2].

3 For an excellent example of what this could look like in research, readers are nudged toward [52]. In this paper, the authors designate a section that details their own experiences with the phenomenon they explain in order to ‘express researcher personality’. In doing so, they embed their personal narratives into the phenomenon — appreciating themselves as agents actively engaged *in* and *with* the research.
